# Sialolithiasis

**Published:** 1904-07-02

**Authors:** 


### SIALOLITHIASIS.

Although in practice salivary calculi are not often met with, yet it is of considerable importance to bear in mind their occasional occurrence, the more so as timely and judicious surgical interference is likely to save the patient from a distressing complaint which otherwise may terminate in disaster. Only too frequently are the submaxillary swelling and other symptoms caused by a calculus impacted in Wharton's duct attributed to dental sepsis or malignant disease, with the result that teeth are extracted without any benefit, or a grave and unwarranted prognosis is given. The submaxillary duct and gland are the most frequent situations of concretions. In 47 cases collected by Dr. Theodore Roberg[1] Wharton's duct was the seat of lodgment in 28, the submaxillary gland in 14, Stensen's duct in two, the sublingual gland in two, the parotid gland in one. This author considers that the presence of a foreign body is one of the contributing causes of calculus formation, and if this be true the relative frequency with which the submaxillary gland and its duct are affected is adequately explained, for foreign bodies are more liable to gain entrance to Wharton's duct than any of the other salivary ducts. But the presence of a foreign body cannot be the sole cause, for as Dr. Roberg points out, extraneous substances are found in the ducts more frequently without any calculous formation. Some additional factor must be present, and this may be bacterial infection. In this connection it will be noted that the parotid duct is the most commonly affected by obvious microbic invasion, and yet is but seldom the site of a concretion; and, indeed, infection with micro-organisms occurs with a frequency out of all proportion to the production of sialolithiasis, so that something in addition to infection must be required. There is, therefore, a good deal of reason to suspect, as well from analogy with other organs as from particular considerations, that the essential requirements for the production of sialolithiasis are prolonged obstruction plus bacterial infection.

Dr. Roberg points out that salivary calculi are composed mainly of calcium carbonate and calcium phosphate; he shows further that these salts are deposited if the solution containing them be rendered more alkaline, and, in the case of saliva, a very small increase of alkalinity is enough to cause precipitation of the calcium salts. By the bacterial decomposition of the salivary proteids ammonia is produced in sufficient quantity to cause deposition of the calcium salts, and in all probability this is the manner in which salivary concretions are formed. The symptoms of sialolithiasis, Dr. Roberg says, are determined by the size of the calculus, its location, and the occurrence of suppuration. In the absence of the last-mentioned phenomenon, a concretion may exist for years without producing much disturbance. The most characteristic symptom of a calculus in Wharton's duct, and usually the earliest, is the so-called "salivary colic," which is characterised by intermittent retention of saliva, and accompanied by more or less pain and discomfort. The accumulation of saliva with the formation of a swelling in the floor of the mouth and in the submaxillary region usually comes on at meal times and remains for one or more hours, and then gradually disappears. It may be possible to cause the retained saliva to escape into the mouth by pressing upon the swelling. The swelling after eating may come and go for years until suppuration takes place. In the latter case the abscess may burst into the mouth, discharging the calculus with the pus, in which case spontaneous cure may result. In other instances a suppurative cellulitis of the neck and face may supervene.

The treatment of salivary calculus in the early stages is to remove the stone, if possible, through the mouth under local anæsthesia. If the concretion be situated in the substance of one of the glands, it must be exposed from the outside. If in such a case a single calculus is found, and the gland is not much changed, the calculus can be removed and the gland left; but if the calculi are multiple and difficult to remove, or if the gland is the seat of miliary abscesses it will be better to extirpate the gland, except in the case of the parotid, where incision, extraction of the calculi, and drainage are all that can be done.

[1] Annals of Surgery, May 1904.

